# Efficacy of neoadjuvant endocrine therapy in patients with poorly differentiated neuroendocrine carcinoma of the breast: A case report

**DOI:** 10.1097/MD.0000000000022652

**Published:** 2020-10-23

**Authors:** Yonglin Zhang, Chao Liu, Chaoting Zheng, Qiaozhen Ren, Qimin Wang, Xinyi Gao, Yushuang He, Jierong Wu, Guanglei Chen, Xuelu Li, Zhenhai Ma

**Affiliations:** a Department of Breast Disease and Reconstruction Center, Breast Cancer Key Lab of Dalian, The Second Affiliated Hospital of Dalian Medical University, Dalian, Liaoning; b Department of Nursing, The Affiliated Hospital of Guizhou Medical University, Guiyang, Guizhou; c The sixth people's hospital of Dalian, Dalian, Liaoning; d Department of Pathology, The Second Affiliated Hospital of Dalian Medical University, Dalian, Liaoning, P.R. China.

**Keywords:** breast cancer, fertility, neoadjuvant endocrine therapy, poorly differentiated neuroendocrine carcinoma

## Abstract

**Rationale:**

Poorly differentiated neuroendocrine carcinoma of the breast is a rare cancer with poor prognosis. There is no standard treatment for the disease. Neoadjuvant therapies and surgery are considered to be the main treatment when the tumor diameter is greater than 5.0 cm. Neoadjuvant therapies include chemotherapy and endocrine therapy. However, the effect of neoadjuvant endocrine therapy is not clear in the disease.

**Patient concerns:**

In August 2014, a 28-year-old premenopausal woman noted a mass that was approximately 3.0 cm∗2.0 cm in size on her right breast with pain. Subsequently, the mass has been always increasing significantly. In August 2015, the mass was approximately 7.0 cm∗5.0 cm in size, accompanied by pain, no nipple retraction and discharge, no orange peel-like skin changes, and no dimples. In addition, she had no salient past history.

**Diagnoses:**

Histopathological examinations by a biopsy with a thick needle (hollow needle) and surgical resection confirmed poorly differentiated neuroendocrine carcinoma of the right breast.

**Interventions:**

First and remarkably, she underwent 3 months of neoadjuvant endocrine therapy (goserelin once every 28 days, and letrozole 10 mg every day). Then, she underwent surgery - stage I breast reconstruction by using prosthesis. Adjuvant endocrine therapy has been used since the operation.

**Outcomes:**

According to response evaluation criteria in solid tumors 1.1, the tumor was shrunk by 78.87% after neoadjuvant endocrine therapy. No salient complications were observed. We have followed her for 48 months, and there are no signs of recurrence and metastasis.

**Lessons:**

Poorly differentiated neuroendocrine carcinoma of the breast is rare and has a poor prognosis. Currently, there is no standard treatment for this disease. Studies show estrogen receptor and progesterone receptor of neuroendocrine carcinoma of the breast are often highly expressed. In the case, it can be observed that estrogen receptor and progesterone receptor are highly expressed. Therefore, neoadjuvant endocrine therapy may be considered in neuroendocrine carcinoma of the breast when the mass is large and the patient refuses neoadjuvant chemotherapy. We hope to provide an attractive evidence for neoadjuvant endocrine therapy of neuroendocrine carcinoma of the breast. However, more cases are still being needed for research.

## 1 Introduction

Neuroendocrine cancer can occur in many organs and systems of human body, such as the digestive system, respiratory system, urinary system, and reproductive system, and it is most common in the digestive system and respiratory system.^[1–4]^ However, neuroendocrine carcinoma of the breast (NECB) is exceedingly rare. In 1977, Cubilla and Woodruff^[5]^ first reported primary neuroendocrine carcinoma of the breast. Then, a study^[6]^ showed that NECB accounts for less than 1.0% of all kinds of neuroendocrine cancer and accounts for 2.0% to 5.0% in all types of breast cancer. Literatures reported that NECB occurs mostly in women, and the age of onset is between 60 and 70 years.^[7,8]^ However, a study indicated that a patient with this tumor was only 13 years old.^[9]^ The finding suggests that this type of breast cancer can also occur in young and middle-aged women. In addition, it used to occur in a man.^[10]^ NECB is a rare type of breast cancer and large-scale studies are not available, so there are no standard treatments currently. The poorly differentiated neuroendocrine carcinoma of the breast is a subtype of NECB with high malignancy and poor prognosis. Therefore, we report a case of poorly differentiated neuroendocrine carcinoma of the breast which was treated successfully by neoadjuvant endocrine therapy, and she successfully gave birth to a baby. Currently, there is no relevant report in English literature, and we hope to provide an attractive evidence for neoadjuvant endocrine therapy of NECB.

## 2 Case report

In August 2014, a 28-year-old premenopausal woman noted a mass that was approximately 3.0 cm∗2.0 cm in size on her right breast with pain. Subsequently, the mass has been always increasing significantly. In August 2015, the mass was approximately 7.0 cm∗5.0 cm in size, accompanied by pain, no nipple retraction and discharge, no orange peel-like skin changes, and no dimples. The physical examination was able to reach a distinct mass located in the outside and lower quadrant of the right breast. The mass was approximately 7.0 cm∗ 5.0 cm ∗ 4.0 cm in size. The texture was hard, the boundary was unclear, the shape was irregular, and the activity was poor, but the skin and chest muscles were not damaged. Axillary lymph nodes cannot be touched.

Ultrasound examination of the breast revealed a large hypoechoic mass in the outside and lower quadrant of the right breast. The mass was approximately 7.1 cm∗5.3 cm∗4.2 cm in size. The boundary was clear, the shape was irregular, the angle was visible, the local burr was visible, and the blood flow signal around the mass was visible (BI-RADS 4B); no enlarged lymph nodes were found under the ultrasound examination. The breast-enhanced magnetic resonance imaging (MRI) showed that a mass shadow was located in the outside and lower quadrant of the right breast. The shape was irregular, the edge was lobulated, the boundary was not clear, the maximum cross-sectional size was approximately 5.7 cm∗3.5 cm. Its T1WI showed a low signal, and T2WI showed a high signal. There was a plate-like higher signal region inside of the mass, and it appeared as fast-in and fast-out enhancement in enhanced scanning imaging. Its center exhibited patchy non-enhanced region, the dynamic enhancement curve was fast-rising and slow-falling type, and the diffusion weighted imaging (DWI) was high signal. A clinical diagnosis of right breast cancer had been made.

In order to clarify the nature of the tumor, she underwent a biopsy with a thick needle (hollow needle). Histopathological examination confirmed poorly differentiated neuroendocrine carcinoma of the right breast. The cancer cells were small in size and showed multifocal coagulative necrosis. There was no clear neurological and vascular invasion. Immunohistochemical staining revealed that tumor cells neuron-specific enolase (NSE) (+++), chromogranin A (CgA) (+++), synaptophysin (Syn) (-),estrogen receptor (ER) 90% medium intensity (+), progesterone receptor (PR) 60% medium intensity (+), human epidermal growth factor receptor-2 (HER-2) (1+) and antigen KI-67 index of 50%. Furthermore, other examinations included head and chest computed tomography (CT), abdominal ultrasound and bone scans, and no signs of metastasis were found in bone, lung, liver, and brain.

**Figure 1:**
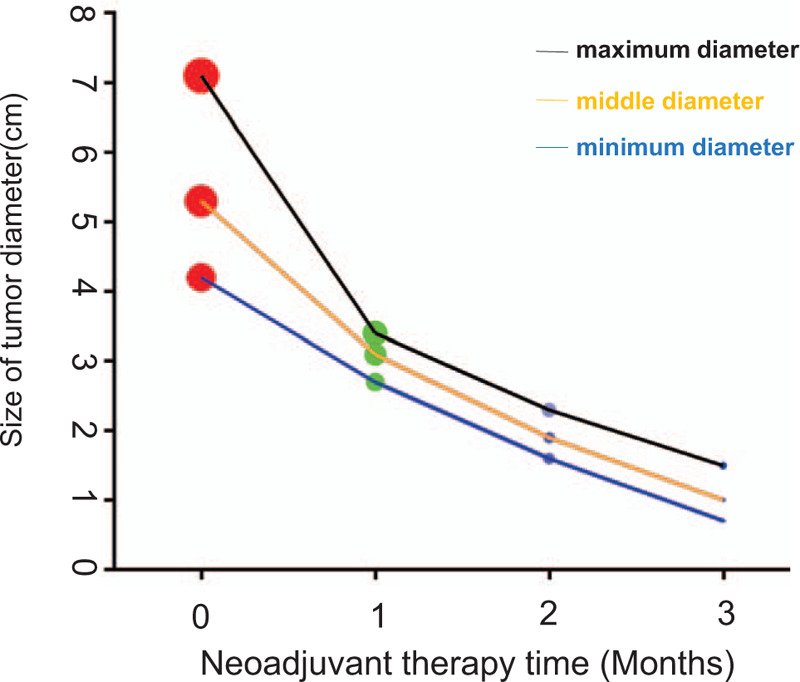
The shrinking tendency of this mass by breast ultrasound images during neoadjuvant endocrine therapy.

**Figure 2:**
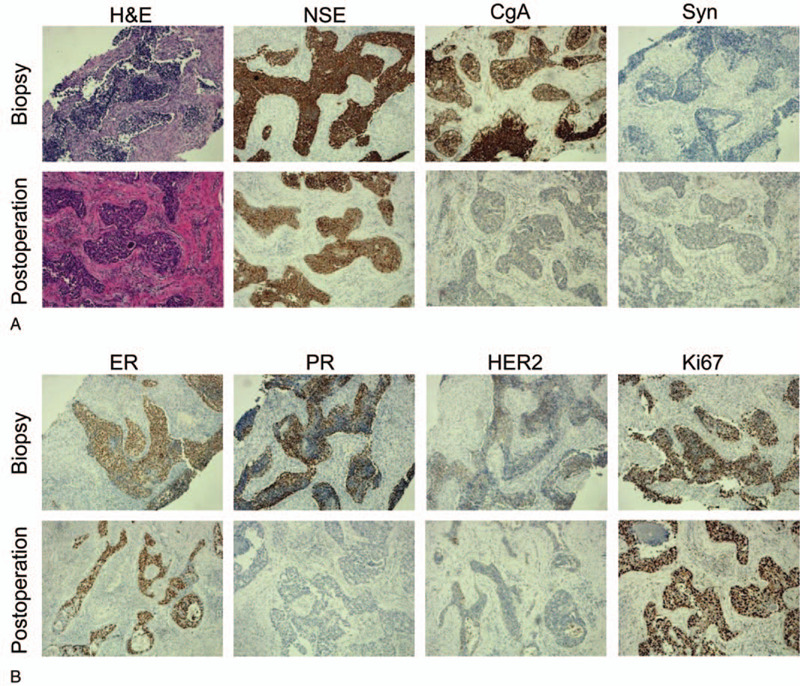
The comparison of pathological and immunohistochemical results of biopsy and postoperation. A. Hematoxyline eosin (H&E) staining and diagnosis-related markers. B. Treatment-related markers.

As the diameter of the mass was greater than 5.0 cm, we recommended that she should receive neoadjuvant chemotherapy. However, she did not agree with our recommendation. Then, she wanted to conserve the breast. According to the result of the STAGE trial^[11]^: preoperative endocrine therapy ovarian function inhibitors combined with aromatase inhibitors was better than ovarian function inhibitors combined with tamoxifen (TAM) in premenopausal women, we recommend that she should receive neoadjuvant endocrine therapy. Ultimately, she underwent neoadjuvant endocrine therapy for 3 months (including goserelin once every 28 days, and letrozole 10 mg every day). No salient adverse events were observed. The curative effect was evaluated by breast ultrasound every month. In November 2015, the breast ultrasound revealed that the mass was approximately 1.5 cm∗1.0 cm ∗0.7 cm in size, the breast-enhanced MRI showed that the mass was approximately 1.8 cm∗1.7 cm in size, and no enlarged lymph nodes were found in both axillary fossea. Ultimately, the shrinking tendency of the mass during neoadjuvant endocrine therapy is shown in Figure 1. According to response evaluation criteria in solid tumors 1.1, the tumor was shrunk by 78.87%, which was a remarkable result. At this time, she changed her previous decision to conserve the breast and underwent surgery - full right breast gland resection and sentinel lymph node biopsy and stage I prosthesis implantation. Postoperative pathology also confirmed poorly differentiated neuroendocrine carcinoma of the right breast (pT1N0M0, Stage I A). Postoperative immunohistochemistry results revealed that ER 50% (+), PR (-), HER-2 (1+), antigen KI-67 index of 60%, NSE (+++), CgA (+), and Syn (+). Ultimately, the comparison of pathological and immunohistochemical results of biopsy and postoperation is shown in Figure 2. After surgery, we still recommended that she should receive adjuvant chemotherapy and radiotherapy. However, she rejected our recommendation again. Ultimately, she continued to receive adjuvant endocrine therapy (including goserelin once every 28 days, and letrozole 10 mg every day).

**Figure 3:**
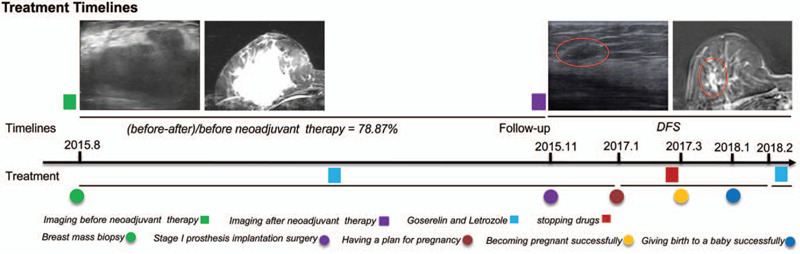
The patient's clinical timeline of diagnosis, treatment, and follow-up.

In January 2017, she planned to become pregnant, and she successfully conceived after stopping the drugs for 2 months. In January 2018, she successfully gave birth to a baby. Both she and her son are recovering well. Our follow-up of this patient lasted for 48 months and the patient's breast shape is now well. Furthermore, no salient adverse events were observed, and there were no signs of recurrence or metastasis. Ultimately, the patient's treatment process is shown in Figure 3.

## 3 Discussion

In 2003, the world health organization (WHO) officially classified NECB as an independent breast disease, and defined it as:

1) a tumor with the same morphological characteristics as the neuroendocrine tumor of gastrointestinal tract or lung,
2) the total number of cells which express neuroendocrine markers are more than 50.0%, excluding metastasis from neuroendocrine carcinoma of other organs. At the same time, according to cell type, tissue level, cell differentiation degree, and mucous production, NECB was divided into 4 subtypes: solid neuroendocrine carcinoma, atypical carcinoid, small cell/oat cell carcinoma, and large cell neuroendocrine carcinoma.^[12]^ In 2012, WHO tumor classification reclassified NECB, and classified the carcinomas with neuroendocrine characteristics into 3 categories:
3) highly differentiated neuroendocrine carcinoma,
4) poorly differentiated neuroendocrine carcinoma (small cell carcinoma),
5) invasive breast cancer with neuroendocrine differentiation.^[13]^ The patient was diagnosed with poorly differentiated neuroendocrine carcinoma of the breast according to the above diagnostic criteria and the latest WHO classification in 2012.

Currently, although studies in large numbers have been focused on NECB, the pathogenesis and etiology of NECB still far from being clarified. A credible study^[14]^ found that the most frequent mutated genes were GATA3, FOXA1, TBX3, ARID1A, PIK3CA, AKT1, and CDH1 in somatic mutations of NECB. Compared with common luminal breast cancers, NECB has a unique repertoire of somatic mutations, has lower TP53 and PIK3CA mutation occurrence, and is enriched with FOXA1, TBX3 mutations, is similar to neuroendocrine neoplasms from other tissues or organs about ARID1A mutations. Then, studies reported that the expression of cytokeratin 7 was strongly positive in NECB, suggesting that mammary stem cells activate under the influence of oncogenic factors and differentiate into endocrine function, which may lead to the occurrence of NECB.^[14,15]^ In addition, androgen can also cause the disease.^[16]^

As the clinical and imaging manifestations of NECB are non-specific, it is extremely difficult to diagnose NECB clinically only by the symptoms, signs, and imaging manifestations. Currently histopathological examination is the gold standard for identifying NECB. There is no doubt that histological and immunohistochemical examinations of specimens after surgical resection and biopsy are quite accurate. In addition, preoperative hollow needle biopsy (histological examination) and fine needle biopsy (cytological examination) are also available. However, Sapino^[17]^ et al. reported that although fine-needle biopsy can accurately diagnose benign and malignant tumors, it is difficult to diagnose NECB due to limited sampling. Therefore, the diagnosis of NECB still requires methods of histopathological examination such as hollow needle biopsy or surgical resection and biopsy. Fine-needle biopsy is not recommended as the diagnostic basis of NECB at present. Hollow needle biopsy can accurately determine nature of breast tumors before surgery, providing a strong basis for feasibility of preoperative neoadjuvant therapy, selection of surgical methods and postoperative adjuvant therapy, contributing to improve compliance of patients and reduce rate of secondary surgery. Therefore, preoperative hollow needle biopsy is more conducive to development of a more reasonable individual treatment program. The patient underwent a hollow needle biopsy and was confirmed as NECB preoperatively. Satisfactory results have been achieved. On the diagnosis and classification of NECB, histomorphology of tumor cells is of great importance, however immunohistochemical detection of neuroendocrine markers is also indispensable. Currently, NSE is recognized as the most common neuroendocrine indicator, but it is not a specific marker of NECB. Therefore, it is not possible that NECB can be diagnosed only by NSE. CgA and Syn are 2 other reliable neuroendocrine markers.^[18]^ The results of neuroendocrine markers of the patient included NSE, CgA, and Syn.

NECB is a rare type of breast cancer, and there are no large-sample studies to indicate its standard treatment. Therefore, strategy formulation of clinical treatment of NECB still refers to the treatment guidelines of general breast cancer for clinicians. The treatment principle of it is still a comprehensive treatment based on surgical treatment. The choice of surgical method is similar to that of general breast cancer, which needs to be considered comprehensively by clinicians according to various factors, such as the patient's gender, age, physical condition, tumor size and location, the proportion between tumor size and breast volume, and the willingness of patients and their families. There are many surgical methods can be selected, including breast reconstruction (including stage I reconstruction and stage II reconstruction), breast-conserving surgery, total mastectomy, and modified radical mastectomy. Radical mastectomy is rarely performed currently. In addition, it is greatly important to exclude distant metastases before surgery, including lung, bone, brain, liver, and soft tissue. In particular, according to the results of the National Surgical Adjuvant Breast and Bowel Project b-06 (NSABP b-06),^[19]^ breast-conserving surgery may be the first choice for patients who meet breast-conserving requirements, especially young women. Its advantages are less traumatic, that it can make the patient maintains well shape of breast, and that it can improve life quality of the patient better. However, patients and their families should fully understand that postoperative radiotherapy is necessary. For evaluation of axillary lymph nodes, sentinel lymph node biopsy should be performed for suitable patients in order to reduce the over-dissection of axillary lymph nodes. It can help reduce the incidence of postoperative complications, such as upper limb lymphedema. Individualized postoperative treatments should be formulated according to the molecular typing of NECB.

For patients of NECB, selection and formulation of radiotherapy and chemotherapy still refer to radiotherapy and chemotherapy criteria of other types of invasive breast cancer. However, compared with general invasive breast cancer, ER and PR of NECB are often highly expressed, and HER-2 is often low-expressed,^[20]^ which proves that the disease is mostly hormone-receptor-dependent type of breast cancer, and endocrine therapy is of great importance. In particular, neoadjuvant endocrine therapy can be selected for suitable patients (elderly patients, patients with poor cardiopulmonary function who cannot tolerate surgery, patients with large tumors or patients with strong desire to preserve breast, and patients who do not agree to neoadjuvant chemotherapy).Then, since HER-2 of NECB is often low-expressed, there is still a lack of relevant evidence-based research of large samples in the biological targeted therapy of NECB with positive HER-2. A small sample of study^[9]^ reported that it was effective and consistent with the effect observed in general invasive breast cancer. Therefore, currently the majority of experts support biological targeted therapy for patients of NECB with positive HER-2. However, the mechanism of function of HER-2 in neuroendocrine also needs to be further explored. No studies have been reported on effect of double-targeted therapy (trastuzumab and partuzumab) for NECB with positive HER-2. The patient's HER-2 is negative and has not been treated with targeted therapy.

About the prognosis of NECB, the existing studies are all reported in small samples, and these studies have not yet reached a unified conclusion,^[20]^ which may be due to the different inclusion criteria of participants in different studies. Then, in prognosis-related studies for different subtypes of NECB, Li^[21]^ et al collected a total of 126 patients of NECB from Chinese journals from 2003 to 2015, and carried out the descriptive statistical analysis. Analysis of prognosis showed that patients with non-small cell carcinoma had a good prognosis, while poorly differentiated neuroendocrine carcinoma (small cell carcinoma) had a poor prognosis. However, the patient did not agree with neoadjuvant chemotherapy and chose neoadjuvant endocrine therapy. After surgery, she also refused chemotherapy and chose to continue endocrine therapy. The patient was followed for 48 months with no signs of recurrence or metastasis, and now she has a well breast shape. Furthermore, she successfully gave birth to a baby. Our case is of great success in neoadjuvant endocrine therapy.

## 4 Conclusions

Poorly differentiated neuroendocrine carcinoma of the breast is a rare disease with no standard treatment. Currently, there is no English literature about neoadjuvant endocrine therapy for poorly differentiated neuroendocrine carcinoma of the breast. Neoadjuvant endocrine therapy may be considered when the mass is large and the patient refuses neoadjuvant chemotherapy. Furthermore, compared with the immunohistochemistry results before and after neoadjuvant endocrine therapy, it can be seen that some changes have occurred in the markers, especially PR. In future word, we will explore the level changes of the markers in disease progression in NECB by basic research. We hope to provide an attractive evidence for future studies.

## Author contributions

All authors were involved in the preparation of this manuscript.

**Collected the data and figures:** Chaoting Zheng, Chao Liu, Qimin Wang, Qiaozhen Ren.

**Performed the operation:** Guanglei Chen, Xuelu Li, Chao Liu, Chaoting Zheng, Xinyi Gao, Yushuang He, Jierong Wu.

**Summarized the data and revised the manuscript:** Yonglin Zhang, Zhenhai Ma.

**Wrote the manuscript:** Yonglin Zhang, Chao Liu.

All authors read and approved the final manuscript.
